# Perception towards the implementation of telemedicine during COVID-19 pandemic: a cross-sectional study

**DOI:** 10.1186/s12913-023-09927-1

**Published:** 2023-09-07

**Authors:** Bayou Tilahun Assaye, Muluken Belachew, Aynadis Worku, Sefefe Birhanu, Ayenew Sisay, Mitiku Kassaw, Habtamu Mekonen

**Affiliations:** 1 Department of Health Informatics, College Medicine and Health Sciences, Debre Markos University, P.O.Box 269, Debre Markos, Ethiopia; 2 Department of Human Nutrition, College Medicine and Health Sciences, Debre Markos University, P.O.Box 269, Debre Markos, Ethiopia

**Keywords:** COVID-19, Perception, Health professionals, Implementation, Telemedicine, Pandemic, Ethiopia

## Abstract

**Background:**

The COVID-19 pandemic has led to a surge in the use of telemedicine as a means of delivering healthcare services remotely. Healthcare providers play a key role in the adoption and implementation of telemedicine for its effectiveness. Despite its benefits, there have been unclear concerns about its effectiveness and acceptance in the process of implementing telemedicine. The objective of the study was to assess health professionals’ perceptions towards the implementation of telemedicine during the COVID-19 pandemic.

**Methods:**

A cross-sectional study design was conducted among eight hundred forty-five study participants from December 2020 to February 2021. A pre-test was performed on 5% of the total sample size, and the quality of the data was ensured by checking its completeness and consistency. Descriptive statistics and bivariable and multivariable logistic regression were used. The Variables with a *P*-value equal to or less than 0.25 in bivariable logistic regression were entered into a multivariable logistic regression, and model fitness was assessed.

**Result:**

The study revealed that 60.9% of professionals had a good perception toward telemedicine implementation, with an 87.2% response rate. Health professionals with IT support staff, ICT training, who use social media platforms regularly, and availability of computer or smartphone within/outside their health facility were 4.7, 3.3, 3.7, and 13.2 times more likely to have a positive association towards telemedicine implementation respectively.

**Conclusion:**

More than half of the health professionals had a good perception of telemedicine. Social media use, ICT training, computer accessibility, and the presence of IT support staff were all found to have positive associations with the telemedicine perception. In the era of the COVID-19 pandemic, the government should take the initiative to strengthen opportunities for health professionals to learn and apply telemedicine in their medical practice by providing ICT training, IT infrastructure and support staff, improving computer access, and recommending health professionals’ positive use of social media in the health facility.

## Introduction

The COVID-19 pandemic severely harmed human ways of life around the world, and poses an unprecedented challenge in healthcare management. It is spread by droplets of saliva or discharge when an infected person coughs or sneezes [1–3].

The impacts of COVID-19 increased the burden on healthcare providers to respond to patient inquiries [4–6]. The global spread of COVID-19 hampered countries’ ability to address and respond to non-communicable diseases, as well as other routine activities [7–10]. To limit its communicability and improve provider preparedness, scholars recommend telemedicine as a preferable channel between health professionals and patients [11, 12]. Moreover, telemedicine overcomes the challenges in the control and prevention of the COVID-19 pandemic during the lockdown and restriction of face-to-face interactions. Health professionals were frightened by COVID-19, and generally, it was difficult to deliver health services during the pandemic [13–15].

Telemedicine has been implemented to promote medication adherence, resulting in fewer readmissions, lower morbidity and mortality, improved access to follow-up care, and high patient satisfaction [16–19]. Even though the patients benefited from increased access to health care, improved healthcare quality, and cost control in the developed countries [20], the perception of telemedicine among health professionals is not identified for long-term telemedicine implementation in resource-limited settings [21, 22].

The use of telemedicine to deliver healthcare services for chronic disease patients has been helpful during this pandemic. According to some studies, health professionals’ positive perceptions towards telemedicine are a critical factor in maximizing telemedicine adoption for communicable disease control and prevention [23, 24].

Social media platforms have been a way to engage with the public to provide insight into healthcare campaigns, a medium for sharing daily events [25], and health professionals have greatly participated in providing COVID-19 information [26–28]. The perceived use of social media by health professionals aids in the delivery of health care services to patients over long distances via telehealth [28, 29]. In the era of the COVID-19 pandemic, different social media platforms such as Facebook, Twitter, and Telegram were accurate reflections of how the disease spread and burden to communities [30]. Social media platforms became a main source of health information about day-to-day events and knowledge-sharing responses to the pandemic [31, 32].

Although, Sharing health information to provide health care services across distance and time has been proposed as a potential solution to the current health care crisis, as it aids in scaling up the health care system and providing health care services to rural and remote areas for disease control and prevention [33].

The potential impact of telemedicine on healthcare delivery is well-known [3]. However, it has failed to sustain and integrate with the healthcare system, particularly in developing countries [34]. To deliver convenient healthcare services to the patient, the healthcare facility needs advanced medical instruments and healthcare services, but some of these are expensive and concentrated in a limited facility [35]. Especially in Ethiopia, the accessibility of healthcare services is costly and risky in the era of the COVID-19 pandemic [34, 36]. Assessing the perception of telemedicine among health professionals is significantly important as a baseline study and full implementation telemedicine system and helps the Ethiopian health system concerning healthcare digitization. Therefore, this study aimed to assess perceptions of health professionals towards telemedicine implementation and associated factors in Ethiopia during the COVID-19 Pandemic.

## Methods and materials

### Study design, setting, and period

An institutional-based cross-sectional study design study was conducted at a governmental health facility among health professionals in Addis Ababa started from December 2020 to February 2021. The study area has a population of more than 4 million. It has ten sub-cities, the capital city of the country, and the AU, and has more than 80 government health facilities in the city and 50 private health facilities. The total number of health professionals in the city is more than 20,000.

### Source and study populations

All health professionals who were working at a governmental health facility were the source population. The health professionals who were working in the selected governmental health facility found in Addis Ababa were the study population.

### Inclusion and exclusion criteria

Health professionals working at government health facilities in Addis Ababa and available during data collection time were included in the study. Health professionals who were seriously ill, unable to respond and positive for COVID-19 were excluded from the study.

### Sample size determination and sampling procedures

The sample size was determined by using the single population proportion formula of 50% of health professionals to determine the perception of telemedicine since there was no previous study done in the same population among health professionals. By considering a 10% non-response rate and stratified the health professionals to their respective departments, so the design effect two and the total sample size was 845 study participants. Were

- ✓ n1 = was the calculated sample size , Z = Confidence interval [95%]
- ✓ P = (Proportion of perception of Telemedicine health professionals =50%
- ✓ 1-p = proportion of No perception of Telemedicine health professionals
- ✓ d = marginal error [5%] and design effect =2
  $$\mathrm{n}1=\frac{{\left(Z\alpha /2\right)}^{2} \mathrm{p}\left(1-\mathrm{p}\right)}{{d}^{2}} =\frac{{\left(1.96\right)}^{2} *0.5 * \left(1-0.5\right)}{{\left(0.05\right)}^{2}} \boldsymbol{=384*2+768*0.1=845}$$

Study participants were selected from ten hospitals, two health centers from each sub-city in the Addis Ababa Administration, and a proportionate allocation from each hospital in the Administration, while health professionals were selected using simple random sampling techniques by a lottery method after being stratified according to their respective professions.

### Operational definition

#### Health professional

Employees with at least a diploma certificate in the health professions who provide healthcare services in the study settings were defined as health professionals [37].

#### Telemedicine system

Using video conferencing, store-and-forward imaging, and wireless communications to diagnose or treat a patient, as well as providing remote patient monitoring services, electronic information, and telecommunication technologies are used to support long-distance clinical health and public health care, patients, and health professionals to prevent and control COVID-19 pandemics [38, 39].

#### Telemedicine

Use teleconsultation, Tele-education, teleradiology, mobile data dissemination, and other telemedicine components with interactive video conferencing online, storing and forwarding, remote patient monitoring applications and other audio platforms to diagnose or treat a patient, patient monitoring services, or consult with other health care providers to prevent and control COVID-19 pandemics, [40, 41].

#### Good perception telemedicine

Study participants who scored above the median in the five-point Likert scale of perception questions were categorized as having a good perception of the implementation of telemedicine, while those who scored below the median were categorized as having a poor perception [42].

### Data collection tool and procedures

A structured administrative questionnaire adapted and modified from various literatures was used to collect the data. The questionnaire was prepared in English and included socio-demographic characteristics of the respondents, basic computer skills and internet use, Knowledge on healthcare digitization, and organizational-related questions. Data were collected using 18 a five-point Likert-scale questionnaire, ranging from 1 to 5, i.e., “1” for strongly disagree, “2” for strongly disagree, “3” for neutral, “4” for agree, and “5” for strongly agree. A minimum of “18” and a maximum of “90” can be scored to assess the study participant’s perception of telemedicine during the COVID-19 Pandemic [43–45]. It was pre-tested for reliability before the conduct of the actual data collection. Four Health informatics professionals collected the data after receiving one-day training on the research’s objective, purpose, and how to conduct the data collection and were guided by a supervisor. Both the data collectors and study participants used COVID-19 protective equipment like masks and sanitizers, keeping a 2-m distance.

### Data quality assurance

The content validity of the questionnaire was checked, and the reliability was calculated using Cronbach’s alpha coefficient (α = 0.79). Before the actual data collection, modifications were made based on the pre-test. The data collectors and the supervisor were selected and trained before participating in the actual data collection process. Creating awareness of the purpose of the study, their rights, and confidentiality issues, sufficient time was given to respondents to read and fill in materials carefully. There was continuous supervision up to the end of data collection. After collecting the data, the supervisor and the investigator checked its consistency and completeness. We used a backup system to minimize data duplication and data loss.

### Data processing and analysis

Data was edited and cleaned, then exported to SPSS- 26 for further analysis and to generate descriptive statistics of the collected data to describe variables in the study using statistical measurements. A binary logistic regression was used to analyze the association between the independent variable and the dependent variable (good or poor perception of telemedicine). Then, if the *P*-Value was 0.2, multivariable logistic regression analysis was carried out to assess the perception of telemedicine. A significant association was interpreted using an odds ratio, a 95% confidence interval, and a *p*-value less than 0.05. The Hosmer–Lemeshow test was used to test model fitness, and model multicollinearity was checked using variance inflation factors (VIF).

## Result

### Socio-demographic characteristics

Seven hundred thirty-seven (87.2% response rate) of the study participants were given written consent to respond to all the questionnaires. Among the study participants, 507 (62.8%) respondents were male. The mean age of the participants was 31.32 + 7.33 SD years, and the majority of the respondents were within the age group of 20–29 years. In terms of educational status, most of the respondents had bachelor’s degrees (51.6%). Regarding the professions of the respondents, 150 (20.4%) were medical doctors and 155 (21.0%) were nurses. The average working experience was 3 + 1.2 SD years, in terms of respondents age, 341( 46.3%) were between the ages of 1 and 5, with 701 (95.1%) using computers, PCs, or smartphones to perform various internet tasks (42.8%) (Table 1).

**Table 1:** Socio-demographic characteristics of the health professional towards telemedicine implementation at a health facility, Ethiopia (*N* = 737)

Variables	Categories	Frequency(#)	Percentage (%)
Gender	Male	507	62.8%
	Female	230	31.2%
Age (Years)	20–29	350	47.4%
	30–39	314	42.5%
	Age > 40	73	9.9%
Professionals	Medical Doctor	150	20.4%
	Nurse	155	21.0%
	Midwifery	71	9.6%
	Pharmacy	84	11.4%
	Medical laboratory	55	7.5%
	Radiology	70	9.5%
	Anesthesia	36	4.9%
	Optometry	41	5.6%
	Psychiatry	41	5.6%
	Others1	34	4.6%
Educational Status	Specialist	80	10.9%
	Resident	44	6.0%
	GP	42	5.7%
	Master’s Degree	166	22.5%
	Bachelor degree	380	51.6%
	Others2	25	3.4%
Work Experience (Years)	< 5	341	46.3%
	6–10	169	22.9%
	11–15	71	9.6%
	> 16	156	21.2%

Others1, HIT Environmental health; Others2, Diploma, PhD

### Basic computer skills and internet use

More than two-third of the 457 (62.0%) respondents had taken basic ICT training, 701 (95.1%) used their computer, laptop, or Smartphone for work, and 321 (43.6%) of study participants searched for information online to provide advice. During the COVID-19 pandemic, 584 (29.4%) of study participants were asked for online advice by patients. 238 (32.3%) of the participants in the study communicated with patients via e-mail or social media (24.3%) (Table 2).

**Table 2:** Basic Computer skill and internet use among health professionals working at the government health facility, Ethiopia, (*N* = 737)

Variable	Categories	Frequency (#)	Percentage (%)
ICT Training	Just an introductory level	457	62%
	Certificate in the ICT	102	13.8%
	Never attended the ICT training	178	24.2%
Do you Have a Computers Or, Pc Or Smartphone?	Yes	701	95.1%
	No	36	4.9%
Tasks performed with their computers or, pc or Smartphones?	Microsoft Office	317	21.0%
	Internet access	647	42.8%
	Entertainment like using social media	492	32.6%
	Others3	54	3.6%
The frequency of searching information from internet?	Always	95	12.9%
	Often	233	31.6%
	Sometimes	321	43.6%
	Rarely	13	1.8%
	Never	75	10.2%
Purposes for searching information online media platform	Obtaining information to give patients	584	29.7%
	Patient consultation	541	27.5%
	Literature search	495	25.1%
	Maintain your knowledge and skills	349	17.7%
How often do you use social media platforms	Always	145	19.7%
	often	127	17.2%
	Sometimes	238	32.3%
	Rarely	128	17.4%
	Never	99	13.4%
Requested by the patient’s online advice during the covid-19 pandemic?	Yes	578	78.4%
	No	159	21.6%

Others3, Zoom meetings, Microsoft teams

### Knowledge on healthcare digitization

**Fig. 1:**
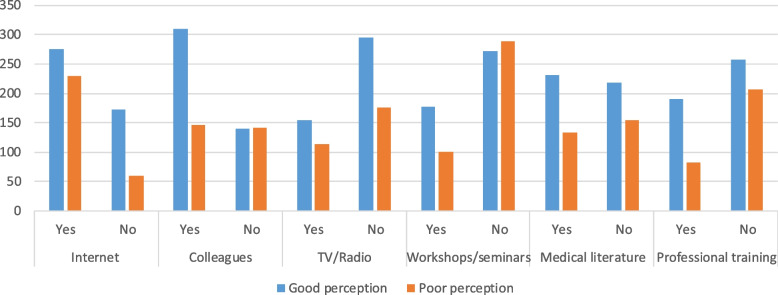
Health professionals’ source of information for telemedicine system

More than half of the participants (497, or 67.4%) were familiar with health care digitations or E-health. The internet was the source of information for those who knew about e-health. 505 (23.6%) and 306 (60.59%) of study participants had visited telemedicine-related websites on the Internet. During the COVID-19 pandemic, 555 (75.3%) of respondents shared information with their friends, consulate healthcare providers, and patients. Although 627 (85.1%) respondents were aware of the factors that impede the use of telemedicine services in the health facility, 576 (25.56%) study participants stated that a lack of telemedicine awareness and 524 (23.25%) a lack of E-health professionals were factors that hampered the implementation and use of telemedicine systems (Fig. 1) (Table 3).

**Table 3:** Knowledge on healthcare digitization and information revolution among health professionals working at health facility, Central, Ethiopia (*N* = 737)

Variable	Categories	Frequency	Percentage
Do you know health care digitization or E-health	Yes	497	67.4%
	No	240	32.6%
Source of information for telemedicine			
Colleagues	Yes	456	61.9%
	No	281	38.1%
Medical literature	Yes	364	49.4%
	No	373	50.6%
Professional training/ conference	Yes	273	37.0%
	No	464	63.0%
Workshop/Seminar	Yes	277	37.6%
	No	560	62.4%
Internet	Yes	505	68.5%
	No	232	31.5%
Radio/TV	Yes	267	36.2%
	No	470	63.8%
Have you ever visited websites related to telemedicine on the internet?	Yes	306	60.59%
	No	199	39.41%
Do you share information with your friends to consulate patients during covid-19?	Yes	555	75.3%
	No	182	24.3%
Type of telemedicine approach do you know?	Store and forward	531	29.3%
	Real-time	333	18.45
	Remote patient monitoring	373	20.6%
	Communication via telephone	553	30.6%
	I don’t know	20	1.1%

### Organizational factors for telemedicine implementation

According to the finding, majority of the study participants 514 (69.7%) had sufficient computers for their work; 525 (71.2%) of the participants had internet access within their health facility, and 241 (45.90%) had both Wi-Fi and broadband types of internet access. Similarly, 555 (75.3%) of the health professionals had an information-sharing culture with other healthcare providers or patients, 461 (62.6%) of the study participants had attended training for E-health or health information revolution, and also 498 (67.6%) study participants had IT, support staff, in their health facility (Table 4).

**Table 4:** Organizational factors for telemedicine implementation among health professionals working at health facilities in Central Ethiopia (*N* = 737)

Variable	Categories	Frequency (#)	Percentage (%)
Accessibility of computers in health facilities	Yes	513	69.7%
	No	223	30.3%
Accessibility of internet health facilities	Yes	525	71.2%
	No	212	28.8%
If you don’t have internet in your health facility, how do you access information	Private Internet	79	37.26%
	Mobile data	127	59.99%
	Others4	6	0.28%
Type of internet access in your health facility	Wi-Fi	182	34.66%
	Broadband	102	19.42%
	Both	241	45.90%
Have you ever taken any training on the e-health or telemedicine system	Yes	461	62.6%
	No	276	37.4%
Do you have ICT supporting staff in your health facility	Yes	498	67.6%
	No	239	32.4%

Others4, Dongle internet from telecoms

### Factors associated with the perception of health professionals towards telemedicine implementation

Computer accessibility, health professionals’ ICT training, frequency of use of social media, and having IT support staff in their health facility, were all significantly associated with the perception of telemedicine implementation among health professionals for the control and prevention of COVID-19. Participants in the study who had sufficient computers were 13.2 times more likely to have a good perception of using telemedicine than those who did not have enough computers in their organization (AOR = 13.21, 95% CI: [5.07–24.05]).

Health professionals in facilities with IT-supporting staff were 4.7 times more likely to have a good perception of telemedicine systems for COVID-19 control and prevention than those in facilities without IT-supporting staff (AOR = 4.42, 95%CI: [CI: 2.958–7.52]). Health professionals certified in ICT were strongly associated with the perception of telemedicine implementation for the control and prevention of COVID-19. Health professionals who had received certification in related ICT areas were 3.26 times more likely to be perceived positively than those who had not (AOR = 3.263, 95% CI: [1.72–6.18]). Another factor influencing health professionals’ perception of telemedicine was their use of social media/Gmail platforms. Health professionals who used social media regularly were 3.7 times more likely to be perceived positively than those who did not (AOR = 3.224, 95% CI: [1.805–7.88]) (Others4, Dongle internet from telecoms, Table 5).

**Table 5:** Bivariable and multivariable logistic regression of factors associated with perception of telemedicine implementation among health professionals working at a health facility, Ethiopia (*N* = 737)

Variables	Categories	Telemedicine Perception		COR (95%CI)	AOR (95%CI)
		Good	Poor		
Gender	Female	150	80	1.3(0.943–1.803)	0.67(0.443–1.029)
	Male	299	208	1	1
Work experience( years)	< 5	225	116	0.703(0.477–1.037)	0.64(0.396–1.016)
	6–10	88	81	1.255(0.810–1.945)	0.773(0.435–1.376)
	11–15	46	25	0.741(0.414–1.326)	0.341(0.196–0.78)
	> 16	90	66	1	1
Computer accessibility	Yes	442	259	7.07(3.05–16.364)	13.210(5.066–24.50)***
	No	7	29	1	1
ICT Training	introductory level	267	190	1.927(1.318–2.817)	1.773(1.024–3.070)**
	Certificate in ICT	52	50	2.604(1.564–4.337)	3.260(1.719–6.180)***
	Never attended ICT training	130	48	1	1
How often do you use social media platforms	Always	82	63	3.035(1.682–5.477)	3.749(1.805–7.788)***
	Often	70	57	3.216(1.761–5.875)	3.328(1.592–6.958)***
	Sometimes	138	100	2.862(1.645–4.981)	3.114(1.592–6.072)**
	Rarely	80	48	2.370(1.291–4.350)	2.071(1.041–4.150)**
	Never	79	20	1	1
Having Information sharing culture	Yes	319	236	0.546(0.376–0.089)	0.572(0.423–1.058)
	No	130	52	1	1
Training on E-health	Yes	145	131	0.572(0.422–0.977)	0.522(0.436–1.030)
	No	304	157	1	1
Availability of IT supporting staff	Yes	320	178	1.533(1.120–2.097)	4.717(2.958–7.522)***
	No	129	110	1	1

*AOR* Adjusted odds ratio, *COR* Crude odds ratio

1 = reference;*** *P*-value = 0.00; ** *p*-value ≤ 0.02

## Discussion

In low-income settings, telemedicine helps users to diagnose and treat patients remotely and offers many advantages to the healthcare system, ranging from immediate access to high-quality healthcare services to reducing healthcare expenses [46]. Providing adequate information about telemedicine technologies to health professionals aids in improving their perceptions.

According to the findings of this study, 60.9% (95% CI: [60.7, 67.4]) of health professionals had a good perception of telemedicine. Perception of e-health is important for the implementation of telemedicine to improve healthcare delivery in rural and remote areas. Currently, the Ministry of Health is focusing on healthcare digitization as one of its top priorities, even though we have limited resources and technological infrastructure to dedicate to the healthcare system [47]. The study is high as compared with the previous cross-section study conducted in Addis Ababa [48]. This could be due to the time lapse between the study’s completion and the Ethiopian government’s increased emphasis on ICT to support the health system.

The finding is also lower than the study conducted in Iran [49]. User motivation and support can help technology become a reality, allowing the system to scale up and the value of health professionals to e-Health to increase [50]. Furthermore, this study differs from a study conducted in Australia [45] that would improve dental practice by improving communication with colleagues and referring new patients. It could be a difference in infrastructure between developing and developed countries.

A study conducted in Saudi Arabia [43] found a high level of perception toward telemedicine when compared with this research finding. It could be due to differences in infrastructure and e-health literacy between the two countries. More than half of the participants were aware of the benefits of telemedicine services. Some scholars indicate that telemedicine can decrease the risk of infection in the hospital, provide adequate treatment and follow-up of patients, and provide a suitable approach for providing remote mental health services [51]. When the participants were aware of the types of telemedicine services available to deliver healthcare services by using telemedicine applications [52]. For both clients and healthcare workers, telemedicine provides continuous access to critical healthcare services in the era of COVID-19 [53].

This study is consistent with a study conducted in Europe, which found that the availability of technology, access to the internet, and a lack of telemedicine training are the most significant factors influencing healthcare provider perception [54]. Besides that, as found in a study conducted in Indonesia, study participants’ lack of internet connectivity was a significant barrier to using telemedicine [44]. A telemedicine training intervention carried out among nursing staff in India demonstrates an effective method of increasing telemedicine awareness and adoption [55]. The pandemic has had a positive impact on the perception of physicians and other stakeholders toward telemedicine and the adoption of this new technology is changing due to the present conduction of COVID-19 [56]. Health professionals who had received ICT training had a positive perception of telemedicine, and the digitization era could help improve their perception.

Another study conducted in developing countries found that many healthcare providers are unable to resolve technical issues caused related to computer systems and ICT networks. As a result, for the telemedicine system to function properly and smoothly, trained and expert personnel are required to establish stable and continuous communication during teleconsultation [57]. It could be due to a lack of technical support, training, or government concern.

According to this finding, frequent use of social media determined whether health professionals were likely to be perceived positively towards telemedicine implementation [25]. The scholars indicated that the COVID-19 medical guidelines have been distributed through social media communication channels such as Facebook, YouTube, or Telegram [58], where health professionals frequently exchange information and disseminate knowledge regarding how to control and minimize the spread of the virus [59].

During lockdown measures at health facilities, physicians have conducted free medical consultations through social media, and researchers disseminate articles published by international authoritative magazines [60]. Sometimes the doctors establish a non-contact doctor-to-patient interactive network platform based on mobile Internet, including social media management, which was used as an important information channel for preventing and responding to the COVID-19 epidemic [61, 62]. Therefore, to scale up and implement the telemedicine system within healthcare facilities, health professionals need ICT training, technical support, and improved internet access to motivate users to use telemedicine.

## Conclusion

More than half of the health professionals had a good perception of telemedicine during the COVID-19 pandemic. The telemedicine concept is more popular among health professionals and has the potential to be fully integrated into the healthcare system. The use of social media, ICT training, computer accessibility, and the availability of IT support staff were all found to be significantly associated with the telemedicine system.

The government should take the initiative to strengthen opportunities for health professionals to learn and apply telemedicine in their medical practice by providing ICT training, and IT support staff, improving computer access, and recommending health professionals' positive use of social media in the health facility to promote the evidence-based use of telemedicine in the era of the COVID-19 pandemic and future outbreaks in health facilities.

### Strengths and limitations of the study

This study has the following strengths. The sample size was large which help to generalize the result. The result of this study will be used as a baseline for other researcher and helps the scalability of health sectors to digital health transformation.

However, the study was not supported by a qualitative study, as we employed a simple random sampling method that may be affected by the sample frame and its lower precision. The study was conducted only in an urban environment, where there is a higher likelihood of perception towards telemedicine.

## Data Availability

All major data have been analyzed and presented in the manuscript.

## Abbreviations

BSC: Bachelor of Science
E-Health: Electronic Health
Epi-info: Epidemiological Information
ETB: Ethiopian Birr
FMOH: Federal Ministry of Health
GP: General Practitioners
HI: Health Informatics
HIT: Health Information Technician
HP: Health professionals
MPH: Master of Public Health
ICT: Information communication technology
IT: Information technology
SPSS: Statistical Package for Social Science
WHO: World Health Organization

## References

[1] Ezhilan M, Suresh I, Nesakumar N (2021). SARS-CoV, MERS-CoV and SARS-CoV-2: a diagnostic challenge. Measurement.

[2] Hassel J, Schütze S, Hagenlocher M. COVID-19 Pandemic.

[3] Lone SA, Ahmad A (2020). COVID-19 pandemic–an African perspective. Emerg Microbes Infect.

[4] Bauchner H, Sharfstein J (2020). A bold response to the COVID-19 pandemic: medical students, national service, and public health. JAMA.

[5] Uy J (2022). The impact of COVID-19 on hospital admissions for twelve high-burden diseases and five common procedures in the Philippines: A National Health Insurance Database Study 2019–2020. Lancet Reg Health-West Pac.

[6] Mehraeen E, et al. Technology in the era of COVID-19: a systematic review of current evidence. 2022;22(4):51–60. 10.2174/1871526522666220324090245 35331123

[7] Akalu Y, Ayelign B, Molla MD (2020). Knowledge, attitude and practice towards COVID-19 among chronic disease patients at Addis Zemen Hospital, Northwest Ethiopia. Infect Drug Resist.

[8] Yadav UN (2020). A syndemic perspective on the management of non-communicable diseases amid the COVID-19 pandemic in low-and middle-income countries. Front Public Health.

[9] Posai V (2021). Assessment of the health-promoting behaviors of hospitalized patients with non-communicable diseases during the second wave of COVID-19. J Multidiscip Healthc.

[10] Mehraeen E, et al. Telemedicine technologies and applications in the era of COVID-19 pandemic: A systematic review. 2023;29(2):14604582231167431. 10.1177/14604582231167431 PMC10116201 37076954

[11] Albarrak AI (2021). Assessment of physician’s knowledge, perception and willingness of telemedicine in Riyadh region, Saudi Arabia. J Infect Public Health.

[12] Almeida F, Santos JD, Monteiro JA (2020). The challenges and opportunities in the digitalization of companies in a post-COVID-19 World. IEEE Eng Manage Rev.

[13] Boehm K, et al. Telemedicine online visits in urology during the COVID-19 pandemic—potential, risk factors, and patients’ perspective. Eur Urol. 2020. 10.1016/j.eururo.2020.04.055 PMC7183955 32362498

[14] Ghai B, Malhotra N, Bajwa SJS (2020). Telemedicine for chronic pain management during COVID-19 pandemic. Indian J Anaesth.

[15] Anthony Jnr B (2021). Integrating telemedicine to support digital health care for the management of COVID-19 pandemic. Int J Healthc Manag.

[16] Kim E, Gellis ZD, Hoak V (2015). Telehealth utilization for chronic illness and depression among home health agencies: A pilot survey. Home Health Care Serv Q.

[17] Kooy MJ (2015). Patients' general satisfaction with telephone counseling by pharmacists and effects on satisfaction with information and beliefs about medicines: Results from a cluster randomized trial. Patient Educ Couns.

[18] Mburu S, Oboko R (2018). A model for predicting utilization of mHealth interventions in low-resource settings: case of maternal and newborn care in Kenya. BMC Med Inform Decis Mak.

[19] Moss HE, Lai KE, Ko MW (2020). Survey of Telehealth Adoption by Neuro-ophthalmologists During the COVID-19 Pandemic: Benefits, Barriers, and Utility. J Neuroophthalmol.

[20] Novara G, et al. Telehealth in urology: a systematic review of the literature. How much can telemedicine be useful during and after the COVID-19 pandemic? Eur Urol. 2020. 10.1016/j.eururo.2020.06.025 PMC7301090 32616405

[21] Lennon MR (2017). Readiness for delivering digital health at scale: lessons from a longitudinal qualitative evaluation of a national digital health innovation program in the United Kingdom. J Med Internet Res.

[22] Zimmerling A, Chen X (2021). Innovation and possible long-term impact driven by COVID-19: Manufacturing, personal protective equipment and digital technologies. Technol Soc.

[23] Rodriguez JA, Clark CR, Bates DW (2020). Digital health equity as a necessity in the 21st century cures act era. JAMA.

[24] Ye Q, Zhou J, Wu H (2020). Using information technology to manage the COVID-19 pandemic: development of a technical framework based on practical experience in China. JMIR Med Inform.

[25] Shamsabadi A (2023). Social media application in education during the COVID-19 pandemic; pros and cons: A systematic review.

[26] Neavel C, Watkins SC, Chavez M (2022). Youth, social media, and telehealth: How COVID-19 changed our interactions. Pediatr Ann.

[27] Farsi D (2021). Social media and health care, part I: literature review of social media use by health care providers. J Med Internet Res.

[28] Machado RA, et al. Social media and telemedicine for oral diagnosis and counselling in the COVID-19 era. 2020;105:104685. 10.1016/j.oraloncology.2020.104685 PMC7151276 32291154

[29] Li H, et al. The establishment and practice of pharmacy care service based on Internet social media: telemedicine in response to the COVID-19 pandemic. 2021;12:707442. 10.3389/fphar.2021.707442 PMC8517072 34658854

[30] Massaad E, Cherfan P (2020). Social media data analytics on telehealth during the COVID-19 pandemic. Cureus.

[31] Zhao S, et al. The opportunities and challenges of telemedicine during COVID-19 pandemic. 2021;13(2):291–298. 10.52586/E885 34937315

[32] Peine A, et al. Telemedicine in Germany during the COVID-19 pandemic: multi-professional national survey. 2020;22(8): e19745. 10.2196/19745 PMC7409912 32568724

[33] Brown NA (1998). The telemedicine information exchange: an online resource. Comput Biol Med.

[34] Dodoo JE, Al-Samarraie H, Alzahrani AI (2021). Telemedicine use in sub-Saharan Africa: barriers and policy recommendations for Covid-19 and beyond. Int J Med Informatics.

[35] Mann DM (2020). COVID-19 transforms health care through telemedicine: evidence from the field. J Am Med Inform Assoc.

[36] Abdela SG (2020). Essential healthcare services in the face of COVID-19 prevention: experiences from a referral hospital in Ethiopia. Am J Trop Med Hyg.

[37] Biruk K, Abetu E (2018). Knowledge and attitude of health professionals toward telemedicine in resource-limited settings: a cross-sectional study in North West Ethiopia. J Healthc Eng.

[38] Fisk M, Livingstone A, Pit SW (2020). Telehealth in the Context of COVID-19: Changing Perspectives in Australia, the United Kingdom, and the United States. J Med Internet Res.

[39] Lonergan PE, et al. Rapid utilization of telehealth in a comprehensive cancer center as a response to COVID-19: Cross-sectional analysis. 2020;22(7):e19322. 10.2196/19322 PMC7340164 32568721

[40] Albarrak AI (2019). Assessment of physician’s knowledge, perception and willingness of telemedicine in Riyadh region, Saudi Arabia.

[41] Bisrat A (2010). Knowledge and perception of health care providers towards telemedicine applications & benefits: a survey from Tikur Anbessa & Nekemete Hospitals.

[42] Ayatollahi H, Sarabi FZP, Langarizadeh M (2015). Clinicians’ knowledge and perception of telemedicine technology. Perspect Health Inf Manag.

[43] Albarrak AI, et al. Assessment of physician’s knowledge, perception and willingness of telemedicine in Riyadh region, Saudi Arabia. J Infect Public Health. 2019. 10.1016/j.jiph.2019.04.006 31060975

[44] Armfield NR, Donovan T, Smith AC (2010). Clinicians' perceptions of telemedicine for remote neonatal consultation. Stud Health Technol Inform.

[45] Estai M, Kruger E, Tennant M (2016). Perceptions of Australian dental practitioners about using telemedicine in dental practice. Br Dent J.

[46] Haleem A (2021). Telemedicine for healthcare: Capabilities, features, barriers, and applications. Sensors International.

[47] Getachew E, Woldeamanuel Y, Manyazewal T (2022). Digital health interventions in the clinical care and treatment of tuberculosis and hiv in central Ethiopia: An initial provider perceptions and acceptability study using the unified theory of acceptance and use of technology model. Int J Mycobacteriol.

[48] Gebre AB (2010). Knowledge and perception of health care providers towards telemedicine applications & benefits: A survey from Tikur Anbessa & Nekemete Hospitals.

[49] Ranjbar H (2021). Iranian clinical nurses’ and midwives’ attitudes and awareness towards telenursing and telehealth: a cross-sectional study. Sultan Qaboos Univ Med J.

[50] Hastall MR, Dockweiler C, Mühlhaus J. Achieving end user acceptance: Building blocks for an evidence-based user-centered framework for health technology development and assessment. in International Conference on Universal Access in Human-Computer Interaction. Springer; 2017.

[51] Elhadi M (2021). Telemedicine awareness, knowledge, attitude, and skills of health care workers in a low-resource country during the COVID-19 pandemic: cross-sectional study. J Med Internet Res.

[52] Wernhart A, Gahbauer S, Haluza D (2019). eHealth and telemedicine: Practices and beliefs among healthcare professionals and medical students at a medical university. PLoS One.

[53] Oyediran KA, Makinde OA, Adelakin O (2020). The role of telemedicine in addressing access to sexual and reproductive health services in sub-Saharan Africa during the COVID-19 pandemic. Afr J Reprod Health.

[54] Hassan A (2020). Global survey on telemedicine utilization for movement disorders during the COVID-19 pandemic. Mov Disord.

[55] Khan I, Dhanalakshami M, Naveena J (2015). Effectiveness of SIM on knowledge Regarding telemedicine among the staff nurses. Int J Nurs.

[56] Sharma M (2020). Tele-ophthalmology: Need of the hour. Indian J Ophthalmol.

[57] Bali S. Barriers to development of telemedicine in developing countries, in Telehealth. IntechOpen; 2018.

[58] Hazza J, Lahrech A (2018). Health care professionals’ social media behavior and the underlying factors of social media adoption and use: quantitative study. J Med Internet Res.

[59] Mulrennan S, Colt H (2020). Medical information and social media in the time of COVID-19. Respirology.

[60] Neely S, Eldredge C, Sanders R (2021). Health information seeking behaviors on social media during the COVID-19 pandemic among American social networking site users: survey study. J Med Internet Res.

[61] Merchant RM, Lurie N (2020). Social media and emergency preparedness in response to novel coronavirus. JAMA.

[62] Merchant RM (2020). Evaluating the potential role of social media in preventive health care. JAMA.

